# A Tonsillar PolyICLC/AT-2 SIV Therapeutic Vaccine Maintains Low Viremia Following Antiretroviral Therapy Cessation

**DOI:** 10.1371/journal.pone.0012891

**Published:** 2010-09-21

**Authors:** Panagiotis Vagenas, Meropi Aravantinou, Vennansha G. Williams, Edith Jasny, Michael Piatak, Jeffrey D. Lifson, Andres M. Salazar, James L. Blanchard, Agegnehu Gettie, Melissa Robbiani

**Affiliations:** 1 HIV/AIDS Program, Population Council, Center for Biomedical Research, New York, New York, United States of America; 2 AIDS and Cancer Virus Program, SAIC-Frederick, Inc., National Cancer Institute, Frederick, Maryland, United States of America; 3 Oncovir Inc., Washington, D. C., United States of America; 4 Tulane National Primate Research Center, Tulane University, Covington, Louisiana, United States of America; 5 Aaron Diamond AIDS Research Center, Rockefeller University, New York, New York, United States of America; University of California San Francisco, United States of America

## Abstract

**Background:**

HIV-infected individuals rely on antiretroviral therapy (ART) to control viral replication. Despite abundant demonstrable benefits, the multiple limitations of ART point to the potential advantages of therapeutic vaccination approaches that could provide sustained host control of viral replication after discontinuation of ART. We provide evidence from a non-human primate model that a therapeutic vaccine applied to the tonsils can maintain low viral loads after cessation of ART.

**Methodology/Principal Findings:**

Animals received 40 weeks of ART initiated 9 weeks after rectal SIVmac239 infection. During ART, animals were vaccinated (or not) with AT-2 inactivated SIVmac239 using CpG-C ISS-ODN (C274) or polyICLC as adjuvants. PolyICLC/AT-2 SIV vaccinated animals maintained viral loads <3×10^3^ copies/ml for up to 16 weeks post-ART, whereas the C274/AT-2 SIV vaccinated and non-vaccinated animals' viremia ranged between 1×10^4^–4×10^5^ copies/ml (p<0.03). Neutralizing Ab activity in plasma was increased by polyICLC/AT-2 tonsillar vaccination under ART, compared to controls (p<0.03). Subsequent vaccination of all animals with polyICLC/AT-2 SIV in the absence of ART did not alter viral loads. Other immune parameters measured in blood and tissues were comparable between groups.

**Conclusions/Significance:**

These results provide support for the potential benefit of mucosally delivered vaccines in therapeutic immunization strategies for control of AIDS virus infection.

## Introduction

Human immunodeficiency virus (HIV-1) infection affects more than 33 million people world-wide, including more than 300,000 children (WHO/UNAIDS). Left untreated, it leads to acquired immune deficiency syndrome (AIDS) and death. More than 2 million people a year die of AIDS-related illness. Whilst a vaccine for HIV remains elusive, major advances in therapies have been made in the last decade.

Highly active anti-retroviral therapy (HAART), introduced in 1996, has so far been very effective in controlling viral replication and preventing the progression of treated patients to AIDS [1]. HAART consists of combination antiretroviral drug therapy, using agents that block different parts of the viral replication cycle. However, despite its impressive demonstrable benefits [2], [3] HAART has major drawbacks. First and foremost, cost and logistical issues have so far precluded delivery of HAART to the majority of infected individuals worldwide who could benefit from this therapy. Drug toxicity and emergence of drug resistant mutant viruses also represent significant challenges [4]. It is also becoming clear that even patients in whom viral replication is effectively suppressed long term can develop serious sequelae [5]. Thus, despite its many benefits, HAART does not represent a definitive approach to the treatment of HIV infection, and harnessing host responses to enhance control of viral replication represents an obvious complementary approach.

Therapeutic vaccination offers the promise of enhancing host antiviral immune responses by immunization under the cover of antiretroviral drug suppression of viral replication, potentially enabling durable control of viral replication even after subsequent discontinuation of HAART. However, most studies of therapeutic vaccination to date in HIV infected subjects have been disappointing [6], [7], [8]. In this non-human primate (NHP) model study, we employ a vaccine immunogen that has shown some promise in initial studies. We used TLR ligands as adjuvants, immunizing via a mucosal route of administration to evaluate the potential of a novel therapeutic vaccination regimen performed during ART to enhance host control of viral replication following discontinuation of antiretroviral drug therapy.

Aldrithiol 2 (AT-2) inactivated SIV has been shown to be effective in inducing responses that can help control SIV infection in macaques [9], [10]. This type of chemical inactivation renders the virus non-infectious by covalent modification of internal virion proteins required for viral replication, whilst preserving the structural and functional integrity of the viral envelope glycoproteins [11], [12], [13]. AT-2-treated viruses interact authentically with DCs [14] and elicit both CD4^+^ and CD8^+^ T cell responses *in vitro* [15]. Moreover, this type of inactivated virus has shown promise in a therapeutic DC-based vaccines [10], [16].

Vaccine immunogens are typically administered with adjuvants in order to stimulate more optimal immune responses associated with protective effects. Regularly used adjuvants range from broadly immunostimulatory compounds, such as alum, to bacterial toxins, such as cholera toxin B. Toll-like receptor (TLR) ligands have recently been used as vaccine adjuvants due to their capacity to mimic components of pathogens and stimulate immune responses. CpG-C immunostimulatory oligonucleotides (CpG-C ISS-ODN) bind to the intracellular TLR9 and have been shown to effectively activate plasmacytoid DC (PDC) and B cell responses [17], [18]. In macaques, CpG-C ISS-ODNs have been shown to induce PDC-derived IFNα and IL-12 production and boost SIV-specific T cell responses *in vitro* [19], as well as B cell proliferation, survival and activation [20]. Injection of CpG-C ISS-ODNs in macaque lymph nodes also activated both DCs and B cells, demonstrating the ability of CpG-C ISS-ODNs to work *in vivo* [21]. Whilst CpG-C ISS-ODNs did not enhance the effect of AT-2 SIV as a preventative vaccine [9], it is possible that their effect might be more pronounced in an already primed, therapeutic vaccine setting. PolyIC is a TLR3 ligand that has been recently shown to block DC-driven HIV replication, via a type-I IFN-dependent pathway [22]. PolyIC has been used in past studies as a vaccine adjuvant [23], [24], [25], [26], [27], [28], [29] and it was shown to be effective in stimulating immune responses. It is thus an attractive candidate for a SIV/HIV vaccine adjuvant.

Even though a therapeutic vaccine does not seek to prevent establishment of infection after mucosal exposure in the manner a preventative vaccine might, for therapeutic immunization mucosal vaccination has distinct advantages over other routes, such as ease of administration and lower cost. In this study, we tested tonsillar therapeutic vaccination with AT-2 SIVmac239 adjuvanted with CpG-C ISS-ODN vs clinical grade polyIC (Hiltonol or polyICLC). We provide direct evidence of improved virus control under ART after tonsillar immunization with AT-2 SIV and polyICLC. This suggests that non-invasive mucosally applied therapeutic vaccines augmented with polyICLC show promise in controlling AIDS virus replication.

## Materials and Methods

### Animals and Treatment

Adult male Chinese Rhesus macaques (*Macaca mulatta*) were housed at the Tulane National Primate Research Center (Covington, LA). All studies and the use of macaques were approved by the Animal Care and Use Committee of the TNPRC (#A4499-01), which has received continued full accreditation by the Association for Accreditation of Laboratory Animal Care (AAALAC #000594). The animals' average age at the beginning of the study was 5 years and their average weight was 10 kg. All animals tested negative for simian type D retroviruses, simian T cell leukemia virus-1, and SIV prior to use. Animals were housed and cared for in compliance with the regulations detailed under the Animal Welfare Act, the Guide for the Care and Use of Laboratory Animals [30], [31]. Animals were monitored continuously by veterinarians to ensure their welfare. The TNPRC Division of Veterinary Medicine has established procedures to minimize pain and distress through several means. Animals were anesthetized prior and during all procedures (10 mg ketamine-HCl/kg). The use of preemptive and post procedural analgesia was required for procedures that would likely cause more than momentary pain or distress in humans undergoing the same procedure. All animals were inoculated rectally with 3×10^3^ TCID_50_ of SIVmac239. ART was given to all animals between 9 and 49 weeks post-infection (40 weeks in total) and consisted of PMPA (20 mg/kg/day) and FTC (40 mg/kg/day), administered subcutaneously. The dosage was halved after 23 weeks of treatment due to the low phosphorus levels in some animals, attributed to potential ART toxicity. During therapy, test animals were vaccinated 4 times, over 2 days each time (i.e. each of the 4 immunizations consisted of 1 vaccination per day for 2 consecutive days, which is the optimal way of delivering polyICLC – A. Salazar, personal communication), with C274/AT-2 SIVmac239 (1.5 mg of C274, 5 µg of p27^CA^ equivalent) or polyICLC/AT-2 SIVmac239 (1 mg of polyICLC/Hiltonol®, 5 µg of p27^CA^ equivalent) administered to the tonsillar tissue in a total volume of 100 µl, applied over the palatine tonsils and the back of the tongue, at 6 week intervals (starting on week 26 post infection). Control animals did not receive any vaccination (adjuvant-only controls groups were not included due to lack of extra animals). 5 weeks after the last immunization, ART was terminated for both vaccines and unvaccinated controls. Table 1 lists all study animals and their respective treatment groups. 20 weeks after ART ended all animals were immunized with polyICLC/AT-2 SIVmac239, 4 times over 2 days, at 6 week intervals (as above).

**Table 1:** Animal treatment details.

Immunization	Animal ID	ART responsive	IFNγ	SIV Ab responses					CD4 counts			
				Plasma	Rectal	Duodenum	Ileum	Jejunum	Pre	Wk 1 post ART	Wk 26 post ART	Final (necropsy)
C274/AT-2 SIV	GJ39	+	+	+	+	+	+	-	850	302	683	1013
	GJ47	+	-	+	+	+	-	+	944	508	**385**	584
	GJ48	+	+	+	+	+	-	-	368	842	**388**	**239**
	GJ50	+	+	+	+	+	-	-	482	1083	**168**	283
	GJ53	+	+	+	+	+	+	-	991	1357	1009	785
	BG93	+*	+	+	+	+	+	+	584	538	672	272
polyICLC/AT-2 SIV	GJ55	+	+	+	+	+	+	+	1020	192	824	1092
	GJ57	+	+	+	+	+	+	n/a	1250	368	708	2091
	CC48	+	+	+	+	+	-	+	390	440	477	235
	DV67	+	+	+	+	+	-	-	121	537	457	598
	DD94	+	+	+	+	+	-	-	351	394	637	502
	GJ97	+*	+	+	+	+	-	-	499	744	**117**	**147**
Control	DA47	+	-	+	+	+	-	-	796	510	788	637
	CM96	+	+	+	+	+	n/a	n/a	912	583	479	651^1^
	CK25	+	+	+	+	+	n/a	n/a	579	290	458	458^1^
	CL68	+	+	+	+	+	n/a	n/a	597	627	**350**	361^1^
	EL02	-	+	+	+	+	n/a	n/a	608	285	n/a	n/a
	GJ65	-	+	+	+	+	n/a	n/a	751	686	n/a	n/a

**Figure 1:**
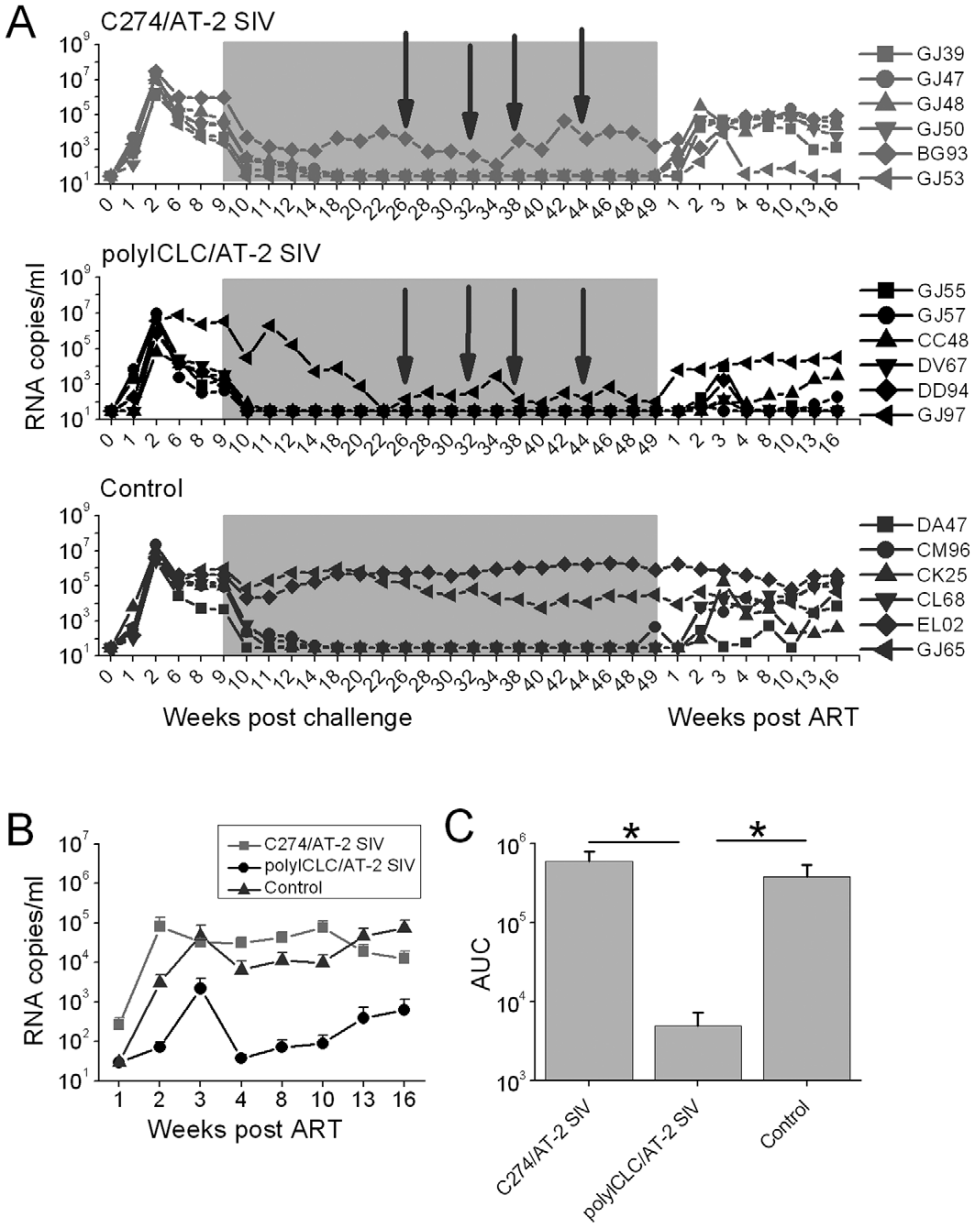
Viral loads of polyICLC-treated animals are significantly lower, after cessation of ART. (A) Plasma SIV RNA copies/ml were determined by PCR. Each symbol indicates an individual animal. The ART treatment period is indicated by the shaded grey box. Arrows indicate immunization time-point. (B) Average (geometric means) viral loads (±SEM) are shown for the ART-responding animals (5 in the C274/AT-2 SIV group, 5 in the polyICLC/AT-2 SIV group and 4 in the control group). Asterisks indicate significant differences between: polyICLC vs both C274 and controls at weeks 2, 4, 8 and 16, p<0.03; polyICLC vs C274 at weeks 3 and 10, p<0.02; polyICLC vs control at week 13, p<0.03. (C) Average Area Under the Curve (AUC) is shown for the ART-responding animals in each group (±SEM). p<0.03 for polyICLC vs C274 and p<0.02 for polyICLC vs control.

*indicates partial ART responsiveness (i.e. viremia >30 copies/ml; see Fig. 1). IFNγ positivity defined as >50 SIV-specific spots (SFC) per 10^6^ cells at >2 time-points. SIV Ab measured in the plasma were IgG, whereas in tissue fluids IgA was measured. Rectal fluid IgA was measured at three time-points (baseline, post-infection/pre-ART and post-ART) and all animals were positive at both post-infection time-points. Intestinal tissue fluid IgA was measured at necropsy. CD4 counts are shown for 4 time-points. Necropsies were scheduled at weeks 65–70 post ART cessation, except control animals that died earlier (weeks 42, 51 post-ART). Animals showing a large drop in CD4^+^ T cells relative to pre-infection values are in bold.

1 Early necropsy due to sickness.

Immune responses were followed by collecting EDTA blood (<10 ml/kg/month) and mucosal (oral and rectal) fluids throughout the study. Mucosal fluids were collected as described previously [9]. Blood, fluids and tissue samples were transported for processing and analysis from the TNPRC to our laboratories at the Population Council by overnight courier service and processed as described previously [9]. Upon study termination or if an animal became ill, animals were euthanized using methods consistent with recommendations of the American Veterinary Medical Association (AVMA) Panel on Euthanasia. Necropsy was performed, when possible.

### Reagents

CpG-C ISS-ODN C274 was provided by Dynavax Technologies (Berkeley, CA). The sequence was: C274 5′-TCGTCGAACGTTCGAGATGAT-3′. PolyICLC (Hiltonol ®) was provided by Oncovir Inc. (Washington, DC). AT-2 SIV (AT-2 SIVmac239 lot numbers: P4001, P4146, P3876, P3778, P3782) and the no virus microvesicle (MV) controls (Lot numbers: P3826, P3971), prepared from the same cell line in which the viruses were grown (SUPT1), were provided by the Biological Products Core, AIDS and Cancer Virus Program, SAIC Frederick, Inc. National Cancer Institute, Frederick (Frederick, MD). AT-2 SIV was used at 300 ng of p27^CA^/ml for all *in vitro* cultures. MVs were normalized to SIV on total protein (300 ng of p27^ CA^/ml equivalent). Concanavalin A (ConA; Sigma, St Louis, MO) was used at 1 µg/ml.

### Cell isolation

Macaque peripheral blood mononuclear cells (PBMCs) were isolated using Ficoll-Hypaque density gradient configuration (GE Healthcare, Sweden). Cells were cultured in complete RPMI 1640 (Cellgro, Springfield, NJ) containing 2 mM L-glutamine (GIBCO Life Technologies, Grand Island, NY) 10 mM HEPES (N-2-hydroxyethylpiperazine-N'-2-ethanesulfonic acid) (GIBCO Life technologies), 50 µM 2-mercaptoethanol (Sigma), penicillin (100 U/ml)/streptomycin (100 µg/ml) (GIBCO Life Technologies) and 1% heparinized human plasma (Innovative Research, Southfield, MI).

Tissue biopsies were placed in RPMI (supplemented as above, but with 10% heat inactivated fetal bovine serum (Mediatech, Manassas, VA) instead of human plasma) containing 200 µg/ml gentamycin (GIBCO) for 1 hour at 4°C. Lymph nodes were cut in 2–4 mm pieces using a forceps and a scalpel and pushed through a 70 µm nylon filter (BD Falcon) with a glass rod. The suspension was spun at 340 g for 10 min. Cells were then resuspended in RPMI (10% FBS) and counted. Jejunum and ileum were washed by spinning at 244 g for 10 min and resuspended in RPMI (10% FBS) containing 0.5 mg/ml Collagenase II (Sigma, St Louis, MO) and 1 mg/ml DNAse I (Roche, Indianapolis, IN) and 1 mg/ml hyaluronidase (Sigma) in a T25 tissue-culture flask (BD Falcon). The tissue was broken up using a forceps and a scalpel and incubated at 37°C for 30 min. The suspension was then passed through a metal sieve using a glass rod. Medium with enzymes, as above, was added to the remaining tissue and incubated at 37°C for a further 30 min. The suspension was passed through a metal sieve again and the filtered suspension from both cases was then filtered using a 70 µm nylon filter. The remaining cell suspension was spun at 340 g for 10 min. Cells were then resuspended in RPMI (1% human plasma) and counted.

### Viral Load and SIV Ab detection

Plasma samples were collected from all animals at all time points of the study, as described previously [9]. SIV RNA was determined by quantitative RT-PCR [32] and SIV-specific Abs were measured by ELISA [33].

Neutralizing Ab activity against SIVmac251 was measured in monkey plasma samples, as described previously [9], with minor adaptations. Briefly, heat-inactivated (56°C, 1 h) plasma was pre-incubated with virus (50 TCID_50_) for 1 h at 37°C before adding 3×10^5^ 174xCEM cells per well (96 well flat bottomed plate). Additional plasma was added on days 3 and 5 of culture. Plasma was tested at 1∶40, 1∶100, 1∶500, 1∶2,500, and 1∶12,500 final dilutions. Cell-free supernatants were collected at day 7 and infection measured by p27 ELISA. Pooled plasma from SIVmac239Δnef/wild type-infected animals (healthy, long-term infected) was used as a positive control. Pooled plasma from uninfected monkeys was used as a negative control alongside a no plasma control.

SIV-specific IgA was measured in rectal and/or intestinal fluids collected at the beginning of the study, at the last time point prior to vaccination (week 26) and at necropsy by ELISA as previously described [34], with minor modifications [9].

### IFNγ ELISPOT

Numbers of IFN-γ spot-forming cells (SFCs) responding to AT-2 SIVmac239 in blood were measured by ELISPOT [19]. ConA was used at 1 µg/ml as a positive control. Medium and MV controls were included for the respective stimuli and background responses to these controls have been subtracted to reveal the specific stimulus-induced responses.

### Flow cytometry

Multi-color flow cytometry was used to characterize leukocyte subsets and polyfunctional T cells in macaque blood and tissues. T cells were characterized using Pacific Blue-conjugated anti-CD3 and PerCP-Cy5.5-conjugated anti-CD4 (clones SP34-2 and L200, BD Biosciences), APC-conjugated anti-CD28 (clone CD28.2, BD Biosciences), PE-conjugated anti-CD95 (clone DX2, BD Biosciences), PE-Cy7-conjugated anti-CD25 (clone M-A251, BD Biosciences), PE-conjugated anti-PD-1 (clone J105, eBioscience), PE-Texas Red conjugated anti-CD38 (clone HIT2, Invitrogen) and APC-Cy7-conjugated anti-CD69 (clone FN50, BD Bioscie3nces). Alexa488-conjugated anti-FoxP3 (clone PHC101, eBioscience) was used to identify Tregs. Intracellular cytokine staining (ICS) was performed as previously described [35]. Cells were stained with anti-CD3 and -CD4 (as above) and FITC-conjugated anti-TNFα (clone MAb11, Biolegend), Alexa700-conjugated anti-IL-2 (clone MQ1-17H12, Biolegend), PE-Cy7-conjugated anti-IFNγ (clone B27, BD Biosciences) and Alexa647-conjugated anti-IL-17 (clone eBio64CAP17, eBioscience).

Appropriate irrelevant specificity isotype Ig negative controls were included in all experiments and typically gave MFIs of <1 log. Samples were acquired on a LSR-II (BD) and analyzed using FlowJo software (Tree Star, OR).

### Reverse Transcriptase Polymerase Chain Reaction (RT-PCR) for immune markers

RNA was extracted from cell pellets (10×10^6^ cells) using the RNeasy Mini Kit (Qiagen, MD) according to the manufacturer's instructions. RNA was immediately quantified using a Nanodrop spectrophotometer (Thermo Scientific, Wilmington, DE) and converted into cDNA using the SuperScript VILO cDNA Synthesis Kit (Invitrogen, Carlsbad, CA). Primers for IFNα, IFNβ, IFNγ, TNFα, IL-2, IL-6, IL-10, IL-12, CCL4, CCL5, CXCL10, FoxP3, IDO and TGFβ were designed using Primer Express (Applied Biosystems, Foster City, CA) and manufactured by IDT (Coralville, IA). The PCR mixture was set-up as follows, per reaction: 12 µl SYBR Green Master Mix (Applied Biosystems, Warrington, UK), 100 nM forward primer, 100 nM reverse primer, 50 ng cDNA and the reaction was made up to 25 µl with distilled water. The program used was 95°C for 10 min, followed by 40 cycles of 95°C for 15 sec and 60°C for 1 min. The reactions were run on an ABI 7700 machine (Applied Biosystems, CA).

### Statistical analyses

Data were analyzed for statistical significance using the Wilcoxon Rank-Sum test in all cases apart from within-group before-after comparisons for neutralizing Ab titers, where the one-sided Wilcoxon Signed-Rank test was used. *p* values <0.05 were taken as statistically significant.

## Results

### Tonsillar polyICLC/AT-2 SIV vaccination maintains low viremia

TLR ligands have been previously shown to be effective inducers of the innate immune response and effective adjuvants. We thus set out to examine two different TLR ligands, polyICLC and CpG-C ISS-ODN C274, as adjuvants in therapeutic vaccination with AT-2 inactivated SIVmac239 in SIVmac239 infected Rhesus macaques. Tonsillar vaccination was used as a model of targeting oral mucosal-associated lymphoid tissue and has been shown by us and other laboratories to be an effective port of entry for immunogens [9], [36], [37], [38].

18 SIV-naïve Chinese Rhesus macaques were inoculated rectally with pathogenic SIVmac239. Infection was confirmed by plasma SIV RNA PCR. Statistical analyses of early plasma viremia (weeks 1–9) showed no differences between the groups, including the setpoint viremia at 9 weeks when ART was initiated. 12 animals were vaccinated 4 times, at weeks 26, 32, 38 and 44 post-infection. 6 received the C274/AT-2 SIV vaccine, whereas the other 6 received the polyICLC/AT-2 SIV vaccine. 6 control animals were not vaccinated. 5 weeks after the final vaccination, ART was withdrawn and the animals were followed up for a further 4 months. Table 1 lists all animals and the treatment they received.

All animals reached peak viremia at 2 weeks post-infection (Fig. 1A). When ART was introduced, viremia was reduced to below detection levels (30 copies/ml) within 5 weeks in almost all animals. There were 4 notable exceptions: 2 monkeys responded to ART partially (one in each vaccine group) and 2 in the control group did not show any response to ART. Data from these animals were excluded from some analyses, in which case it was indicated that only the good ART responders (i.e. animals that achieved and maintained viremia of <30 copies/ml; referred to as ART responders for simplicity) were included. Due to the small numbers of animals we were unable to make significant comparisons between the ART responders and partial or non responders.

Vaccination did not have a demonstrable effect on viral load during the time animals were receiving ART as animals had suppressed plasma viremia to below measurable levels prior to the first immunization (Fig. 1A). Upon cessation of therapy, there was a similar rebound (1–8×10^4^ RNA copies/ml at peak) in the viral loads of all ART-responding animals in the control and C274/AT-2 ISV vaccine groups. Interestingly plasma viremia in the C274/AT-2 ISV vaccine group was on average higher and peaked earlier than that observed for control animals (Fig. 1B), though that difference was not statistically significant at any time-point. In contrast, ART-responding polyICLC/AT-2 SIV vaccinated animals had significantly smaller viral rebounds and all peaked at <3×10^3^ RNA copies/ml 3 weeks after stopping ART (Fig. 1B). All animals were followed to 16 weeks post-ART. C274/AT-2 SIV vaccinated animals and controls maintained high viremia, whereas polyICLC/AT-2 vaccinees maintained lower viremia, with 2 animals having undetectable viral load (<30 copies/ml). Looking at ART-responding animals, at weeks 2, 4, 8 and 16 post-ART polyICLC/AT-2 SIV vaccinated animals had significantly lower viremia than both C274/AT-2 SIV animals and controls (p<0.03 for both). At weeks 3 and 10 post-ART, polyICLC/AT-2 SIV vaccinated animals had significantly lower viremia than C274/AT-2 SIV vaccinees (p<0.02) and at week 13, polyICLC/AT-2 SIV animals had significantly lower viremia than controls (p<0.03). The areas under the curves (AUC) of the viral loads between 1 and 16 weeks post-ART (shown in Fig. 1B) were calculated for each animal and the average for each group is shown (Fig. 1C). Average AUC for the polyICLC/AT-2 SIV animals was significantly lower than C274/AT-2 SIV (p<0.03) and controls (p<0.02).

**Figure 2:**
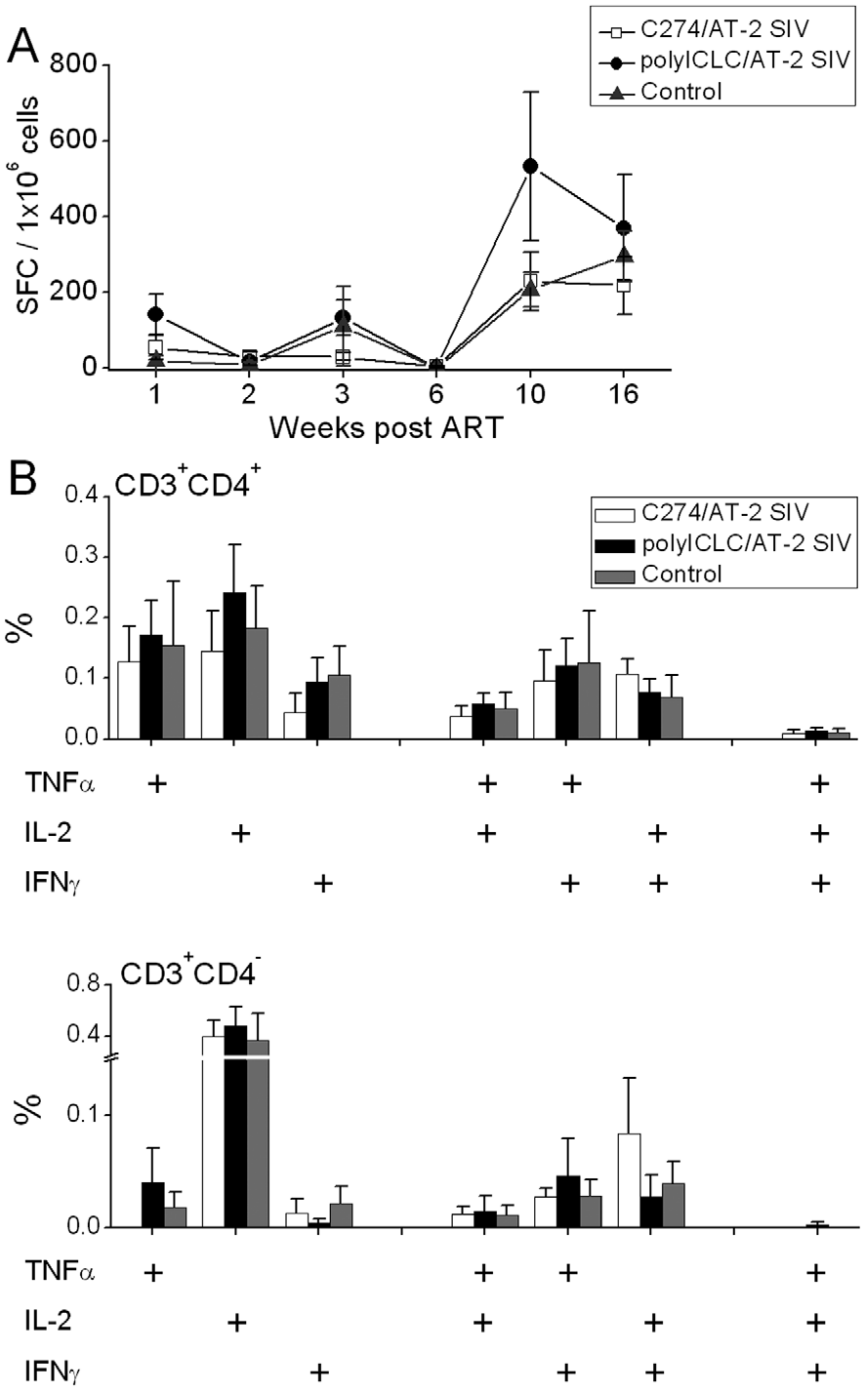
Similar SIV-specific responses in immunized and control animals. (A) Average SIV-specific (to AT-2 SIV) IFNγ ELISPOT responses are illustrated (±SEM). SIV specific responses were determined by subtracting the appropriate MV-induced background. (B) Percentage of cytokine producing (IFNγ, TNFα, IL-2 and combinations) CD3^+^CD4^+^ (upper panel) and CD3^+^CD4^−^ (lower panel) cells were measured in PBMC, by flow cytometry at weeks 17–18 post-ART. Results shown in panels A and B (±SEM) are for 5 animals in the C274/AT-2 SIV group, 5 in the polyICLC/AT-2 SIV group and 4 in the control group.

SIV-specific IFN-γ responses were negligible during ART, increasing post ART in all groups. Comparable levels of SIV-specific T cell responses (normalized by subtracting any background in response to MVs) were detected in the blood of the three treatment groups, by IFN-γ ELISPOT (Fig. 2A) and ICS for TNFα, IFNγ, and IL-2 after discontinuing ART (Fig. 2B). CD4 counts, as well as plasma and rectal fluid SIV-specific binding Abs levels were also comparable across the 3 groups over time (Table 1).

**Figure 3:**
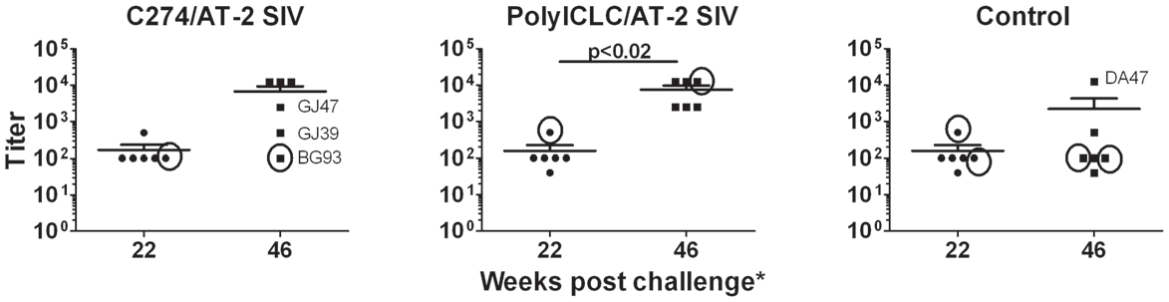
PolyICLC/AT-2 SIV vaccination increases neutralizing Ab activity. Neutralizing Ab titers in the plasma were determined before and after vaccination (vaccination on weeks 26, 32, 38, and 44 post infection). Levels were measured in samples taken 22 weeks after infection and 46 weeks post infection for all animals except CK25 (week 42) and EL02 and GJ65 (week 49) (asterisk). The titer (the last dilution tested at which infection was blocked) is shown for each animal and the mean for each group is marked by the horizontal bar. The circled symbols denote the animals that were ART partial or non-responders. Animal ID numbers are indicated for animals with outlier values relative to the rest of their respective groups.

Neutralizing Abs against SIVmac251 were detected in the blood of all animals post-infection. No differences were observed between animals assigned to the different treatment groups prior to vaccination. Following vaccination with polyICLC/AT-2 SIV under ART, neutralizing Ab titers increased ≥25-fold in all (6 of 6) vaccinees (Fig. 3, p<0.02 relative to pre vaccination). Similar increases were seen in 4 out of 6 C274/AT-2 SIV vaccinees, but this was not significant (p<0.07 relative to pre vaccination). In contrast, only 1 (DA47) of the 6 non-vaccinated control animals had an increase in neutralizing Ab titer over time. Notably, this was the animal that best controlled virus in that group upon cessation of ART (Figure 1). Only the neutralizing Ab titer of the polyICLC/AT-2 SIV vaccinated animals post vaccination was significantly higher than the levels seen in the non-vaccinated controls at the same time post infection (p<0.03).

The major histocompatibility complex (MHC) profile of each animal was determined to ascertain if the animals with the reduced viremia in the polyICLC-treated group had MHC alleles associated with control of viral replication. The following molecules were tested for: Mamu-A*01, Mamu-A*02, Mamu-A*08, Mamu-A*11, Mamu-B*01, Mamu-B*03, Mamu-B*04, Mamu-B*08, Mamu-B*17 and Mamu-DRB*w201. Only 3 animals (GJ48 in the C274/AT-2 SIV group, GJ57 in the polyICLC/AT-2 SIV group and DA47 in the control group) carried the B*03 and/or B*04 alleles, which have been associated with slow disease progression.

### Late immunization with polyICLC/AT-2 SIV in the absence of ART does not affect viremia

Having followed the animals for 4 months after cessation of ART and established that the polyICLC/AT-2 SIV vacinees showed better control of viral replication than the other groups, we evaluated the effects of subsequent immunization with the polyICLC/AT-2 SIV vaccine in the absence of ART in the animals initially vaccinated with polyICLC/AT-2 SIV, as well as the C274/AT-2 SIV vaccinated and control animals. Animals in each original treatment group were immunized with the polyICLC/AT-2 SIV vaccine, as described for the initial vaccinations. The two control animals (EL02 and GJ65) that had not responded to ART were excluded, having already been euthanized. All animals received polyICLC/AT-2 SIV at 20, 26, 32 and 38 weeks post-ART and followed up for 3 months after the final immunization.

**Figure 4:**
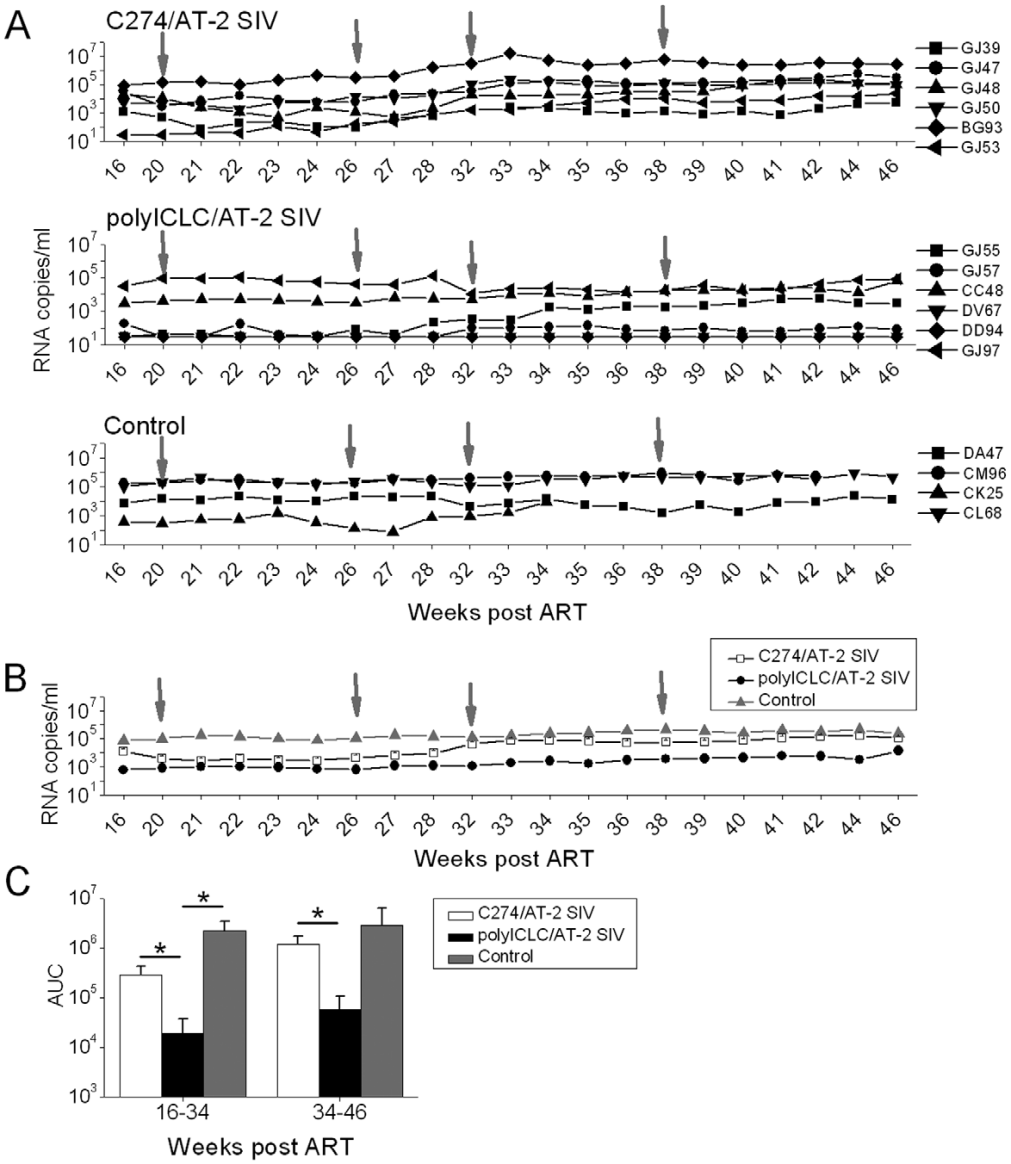
Viral loads are not impacted by late immunization with polyICLC/AT-2 SIV without ART. (A) Plasma viral loads were determined before, during and after treatment. Each symbol indicates an individual animal. Arrows indicate immunization time-point. (B) The average viremia (geometric means) for each group (±SEM) is shown. Asterisks denote the statistically significantly lower virus levels in the polyICLC group compared to the original C274 and/or control groups; p<0.04 for polyICLC vs C274 at weeks 23–46, p<0.03 for polyICLC vs control at weeks 20–34, for good ART responders. (C) Average AUC is shown for each group (±SEM) for the time period shown in panel A. Asterisks mark the statistically significant differences: polyICLC vs C274 at weeks 16–34, p<0.03; polyICLC vs control at weeks 16–34, p<0.03; polyICLC vs C274 at weeks 34–46, p<0.05. Each group's original treatment under ART is indicated in each panel.

Plasma viremia appeared unaffected by the polyICLC/AT-2 SIV immunizations in the absence of concomitant ART (Fig. 4A). In the original polyICLC/AT-2 SIV group, average viral loads remained approximately 2×10^3^ RNA copies/ml for over 5 months after cessation of ART vs 5×10^4^ and 2×10^5^ RNA copies/ml in the original C274/AT-2 SIV and control groups respectively (Fig. 4B). The average viremia of ART responders in the original polyICLC/AT-2 SIV group was significantly lower than the original controls (p<0.03) and than the original C274/AT-2 SIV group (p<0.04), at all time-points up to week 34. Between weeks 34 and 46, statistical significance was only achieved between the original polyICLC/AT-2 SIV and the C274/AT-2 SIV group (p<0.04) and not with the control group, due to the low number of animals remaining in the latter group. These findings correspond to an AUC analysis (Fig. 4C). SIV DNA levels were also measured in the lymph nodes at necropsy with no significant differences observed between groups (data not shown).

### PolyICLC-induced immune responses and long term virus control

**Figure 5:**
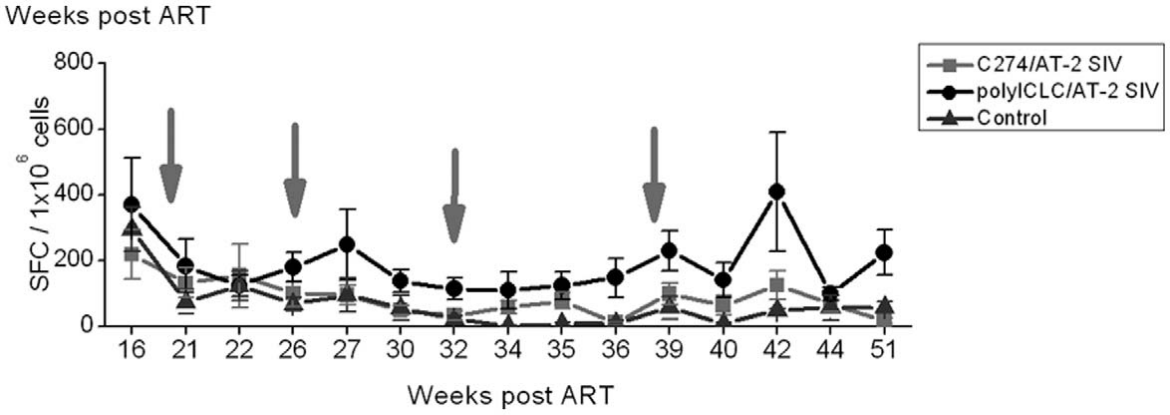
SIV-specific T cell responses were similar across groups. The average SIV-specific (to AT-2 SIV) IFNγ ELIPSOT responses during the vaccination are illustrated (±SEM). SIV specific responses were determined by subtracting the appropriate MV response.

More extensive examination of the adaptive immunity was carried out in the hope of identifying the responses involved in the improved long term control of virus replication in the original polyICLC/AT-2 SIV vacinees. In all animals the frequency of SIV-specific IFNγ producing cells measured in blood was unaltered by the polyICLC treatment in the absence of ART (Fig. 5). Although the responses in the original polyICLC group were often higher than those in the other groups, this was not statistically significant at any time-point. Since the polyICLC/AT-2 SIV vaccination under ART resulted in greater neutralizing Ab responses (Fig. 3), we measured whether neutralizing Ab responses were further boosted in all three groups after polyICLC/AT-2 SIV vaccination without ART. There were no significant changes to the neutralizing Ab levels after poly ICLC/AT-2 SIV vaccination without ART.

We also examined the immune responses by ICS (Tables 2, 3) and RT-PCR (Fig. S2), as well as the distribution of CD25^+^FoxP3^+^ Tregs, CD95^−^ naïve, CD95^+^CD28^+^ central memory and CD95^+^CD28^−^ effector memory CD4^+^ and CD4^−^ T cell subsets in the in blood, lymph nodes and gut tissues (ileum and jejunum), at necropsy. CD38, CD69 and PD1 expression was investigated (blood and tissues) as a measure of T cell activation (Fig. S1). There were no detectable differences in the percentages of T cell subsets and activated T cells between the two treatment groups. TNFα, IFNγ, IL-2 and IL-17 were measured by ICS. SIV-specific single and double cytokine-producing cells were detected in most animals (Tables 2, 3). No triple or quadruple cytokine-producing “polyfunctional” CD4^+^ or CD8^+^ T cells were detected at this time-point. Similarly, there were no differences between the IFNα, IFNβ, IL-6, IL-12p35, FoxP3, IDO and TGFβ RNA levels detected in lymph nodes and jejunum of the two groups (Fig. S2A). TNFα, IFNγ, IL-2 and CCL5 appeared to be marginally elevated in the polyICLC/AT-2 SIV group (Fig. S2B), although the differences seen were not statistically significant. There was no clear correlation between cytokine expression and plasma viral load identified. Therefore, while polyICLC/AT-2 SIV therapeutic vaccination under ART helped control viremia upon removal of ART, the immune responses presumably underlying this enhanced control remain unidentified.

**Table 2:** Limited long-term cytokine/chemokine expression detected across groups (I).

		CD4^+^									
		TNFα	IL-2	IFNγ	IL-17	TNFα + IL-2	TNFα + IFNγ	TNFα + IL-17	IL-2 + IFNγ	IL-2 + IL-17	IFNγ + IL-17
GJ39	PBMC	+	-	+	-	-	+	-	-	-	-
	LN	+	-	-	+	+	+	+	+	-	+
GJ47	PBMC	-	-	-	-	-	-	-	-	-	-
	LN	+	-	-	-	+	-	-	-	-	-
GJ48	PBMC	+	-	+	+	-	+	+	-	+	+
	LN										
GJ50	PBMC	+	--	+	-	+	+	-	+	-	-
	LN	+	+	+	+	-	-	-	+	-	-
GJ53	PBMC	+	+	+	+	+	+	+	-	+	+
	LN	+	+	-	-	+	-	-	-	-	-
BG93	PBMC										
	LN	-	-	+	-	-	-	-	-	-	-
GJ55	PBMC	+	+	+	+	+	+	+	+	+	+
	LN										
GJ57	PBMC	+	-	+	-	-	+	-	+	-	-
	LN	+	-	+	-	-	-	+	-	-	-
CC48	PBMC										
	LN	+	-	-	-	-	-	-	-	-	-
DV67	PBMC	+	-	-	-	-	-	-	-	-	-
	LN	+	+	-	+	+	+	+	+	+	+
DD94	PBMC	-	-	-	-	-	-	-	-	-	-
	LN	+	+	+	+	-	+	+	+	-	+
GJ97	PBMC	-	-	-	-	-	-	-	-	-	-
	LN	+	+	+	+	+	+	+	+	+	+
DA47	PBMC	+	+	+	+	+	+	+	+	+	+
	LN	+	+	-	+	-	-	+	-	-	-

The expression of four cytokines was measured at the end of the study (week 46 post ART) by ICS of PBMC and lymph node (LN) cells. An average value of >0.01% was taken as a positive. Plus-signs indicate positivity, minus-signs indicate negativity, whereas white cells indicate that a value was not determined for that condition. Single and double cytokine combinations are shown. No triple or quadruple cytokine producing cells were detected. SIV-specific responses were measured by subtracting the corresponding MV response. CD4^+^ T cell cytokine production is shown.

**Table 3:** Limited long-term cytokine/chemokine expression detected across groups (II).

		CD4^−^									
		TNFα	IL-2	IFNγ	IL-17	TNFα + IL-2	TNFα + IFNγ	TNFα + IL-17	IL-2 + IFNγ	IL-2 + IL-17	IFNγ + IL-17
GJ39	PBMC	+	-	-	-	-	-	-	-	-	-
	LN										
GJ47	PBMC	+	+	-	-	-	+	-	-	-	-
	LN										
GJ48	PBMC										
	LN										
GJ50	PBMC	+	-	-	-	-	-	-	-	-	-
	LN	-	-	-	+	-	-	+	-	+	-
GJ53	PBMC	+	+	-	+	+	-		-	+	-
	LN	+	+	+	+	+	+	+	+	+	+
BG93	PBMC	-	-	+	-	-	-	-	-	-	-
	LN	+	+	+	+	-	+	+	-	+	+
GJ55	PBMC	+	+	+	-	+	+	+	+	-	+
	LN										
GJ57	PBMC	-	-	-	-	-	-	-	-	-	-
	LN	-	-	-	-	-	-	-	-	-	-
CC48	PBMC										
	LN	+	-	-	+	-	-	+	-	-	-
DV67	PBMC	+	+	-	+	+	-	+	-	+	-
	LN	+	+	-	-	+	+	+	-	+	+
DD94	PBMC	+	-	-	-	-	-	-	-	-	-
	LN	+	+	-	-	+	+	+	+	+	+
GJ97	PBMC	-	-	-	-	-	+	+	+	-	+
	LN	-	-	-	-	+	+	-	+	-	+
DA47	PBMC	+	+	+	+	+	+	+	+	+	+
	LN	+	-	-	-	-	-	-	-	-	-

The expression of four cytokines was measured at the end of the study (week 46 post ART) by ICS of PBMC and lymph node (LN) cells. An average value of >0.01% was taken as a positive. Plus-signs indicate positivity, minus-signs indicate negativity, whereas white cells indicate that a value was not determined for that condition. Single and double cytokine combinations are shown. No triple or quadruple cytokine producing cells were detected. SIV-specific responses were measured by subtracting the corresponding MV response. CD4^−^ T cell cytokine production is shown.

## Discussion

This study aimed to investigate the combination of AT-2 inactivated SIV with 2 TLR-binding adjuvants, C274 and polyICLC, as a mucosal therapeutic vaccine for SIV-infected macaques. We have previously shown AT-2 SIV to be partially effective in preventing SIV infection in macaques [9]. C274 did not enhance the immunogenicity of AT-2 SIV in the preventative vaccine setting, but in infected, SIV-primed macaques it was possible that it would be a more potent adjuvant when boosting existing immunity. PolyIC was shown to effectively block HIV replication in DCs *in vitro* [22], to upregulate CD80 expression on blood mDCs *in vivo* following tonsillar application (E. Jasny and M. Robbiani, unpublished data) and has been shown to be an effective adjuvant [23], [24], [25], [26], [27], [28], [29]. Therefore, we decided to also test the effectiveness of polyICLC (Hiltonol®), as an adjuvant in a separate group of animals.

ART consisted of a two-drug regimen that has been previously shown to be partially effective in reducing plasma viremia [39]. In this case, it was successful in reducing viral replication below the limit of detection in all but four animals. The success in controlling viremia is likely due to the Chinese origin of the macaques (vs. Indian macaques used elsewhere). The lower levels of viremia at the time of initiation of ART contribute to improved virologic responses to treatment, but are also a better match for the levels of viremia typically seen in untreated HIV infected humans [40]. Statistical analyses of early plasma viremia did not reveal significant differences between the groups prior to ART and vaccination. The effectiveness of the two-drug (PMPA/FTC) ART in reducing viral load suggests the usefulness of this model for similar studies in the future.

After the test group animals were vaccinated four times during ART, therapy was withdrawn. As expected from previous studies in macaques [41] as well as humans [42] viremia in the non-vaccinated control animals quickly rebounded to the levels seen before treatment. Similar results were seen in the C274/AT-2 SIV vaccinees. However, in the polyICLC/AT-2 vaccinated animals, after a small spike of viral replication, plasma viremia appeared to be successfully controlled for at least 4 months. In fact, in two out of the five animals that responded to ART, viremia was under the limit of detection for the entire period (almost 1 year post-ART). Thus, polyICLC/AT-2 SIV vaccination resulted in significantly lower virus levels after ART. C274, was not effective as an adjuvant in this case, as in the preventative vaccine setting [9]. A recent study that used a CpG-B ISS-ODN in combination with AT-2 SIV as a therapeutic vaccine, also failed to show efficacy in controlling plasma viremia, despite augmented SIV-specific IFNγ responses [43]. These results suggest that therapeutic vaccination of SIV-infected macaques undergoing ART with polyICLC/AT-2 SIV is effective in the post-therapy control of viral replication. As a result, future studies will need to examine the effect of AT-2 SIV vs. the polyICLC adjuvant alone (which was beyond the scope of this study due to the limitation in animal numbers).

Since the polyICLC/AT-2 SIV vaccination showed promise when administered under ART we were interested to assess how it performed in the absence of ART. Unfortunately, polyICLC/AT-2 SIV vaccination did not reduce plasma virus levels in any of the groups in the absence of ART. However, there were no adverse affects of the vaccination either. SIV-infected Rhesus macaques develop symptoms of simian AIDS relatively rapidly [44]. The fact that these animals remained healthy for up to one year after ART was ended, suggests that the polyICLC/AT-2 SIV vaccinations may have contributed to some degree of viral control in this setting too. However, we were unable to include animals that did not receive the additional polyICLC/AT-2 SIV treatment to know if this had any impact on controlling progression.

We attempted to determine the correlates of immune control of viral replication after the effective vaccination under ART as well as after subsequent vaccination without ART, measuring a variety of adaptive and innate responses in blood and tissues, but most responses were comparable between groups at the time-points measured. However, we were able to detect significantly higher neutralizing Ab activity in polyICLC/AT-2 SIV vaccinees, after the end of ART, which may account for some of the protective effect of the vaccine. It is well established that high neutralizing Ab activity is inversely correlated with plasma viremia [45]. Although multiple cytokine producing “polyfunctional” cells have been shown to play a key role in anti-SIV immunity [46] we did not detect them in the polyICLC/AT-2 SIV vaccinated animals. Additional studies are needed to clarify how the polyICLC/AT-2 SIV-induced virus control is being mediated (e.g., innate and/or adaptive responses not detected or measured herein, monitoring at earlier time points during vaccination).

The role of Tregs in HIV infection is uncertain. They have been shown to suppress HIV-specific T cell responses [47] but on the other hand their numbers decline as disease progresses [48]. A recent study has shown that CD4^+^ Tregs can suppress CD8^+^ T cell responses after therapeutic vaccination [49]. CD8^+^ Treg expansion has been observed concurrently with reduced viral control [50]. In our study, small numbers of CD4^+^ Tregs were identified in the gut of animals, but without significant differences between the test groups. CD69 and CD38 expression was high and similar between groups, consistent with immune activation. Programmed death 1 (PD-1) is a T cell inhibitory receptor [51], [52] that has been associated with T cell exhaustion and failure to contain viral infections, including HIV [53] and SIV [54]. Blockade of PD-1 has been associated with enhanced CD8^+^ T cell response [55], [56]. A recent study, however, suggested that increased PD-1 expression on SIV-specific CD8^+^ T cells should be viewed as a sensitive indicator of low-level ongoing viral replication [57]. PD-1 expression was higher in CD4^+^ T cells from the polyICLC/AT-2 SIV group (although this was not significantly different to the C274/AT-2 SIV group), both in the blood and in lymph nodes. Given that we observed the lowest viral loads in this group and the fact that cytokine production was measured in these cells, it would be unlikely that this result represents dysfunctional T cells. It is important to note that these analyses were only made at the end of the study and more extensive monitoring at earlier time points might reveal insight into the polyICLC/AT-2 SIV-mediated control.

Another level of immune control might be dictated by the MHC allele of individual monkeys. Certain macaque MHC alleles, like Mamu-A*01 [58], B*08 [59] and B*17 [60], lead to better control of SIV infection and disease progression. Others, such as Mamu-DQB1*0601 [61], are associated with rapid disease progression. Genotyping identified Mamu-B*03 and B*04, associated with slow disease progression [62], [63], in 3 macaques, one from each group. This suggests that they do not play an important role in this setting. It should be mentioned however, that previous studies on protective alleles were performed on Indian rhesus macaques and such alleles specific to Chinese macaques are yet to be defined.

Previous studies demonstrated beneficial therapeutic vaccination for SIV infection, administered intramuscularly [64], [65], [66]. Some have also demonstrated vaccine-induced immune responses, but no control of post-ART viremia [43], [67]. Herein we provide the first evidence for the effectiveness of a mucosal therapeutic vaccine for SIV with polyICLC as the adjuvant. Moreover, we documented better control of viremia for a longer period of time post-ART compared to previous studies. This strategy was effective in boosting the animals' immune system to control plasma viremia to remarkably low levels – in some cases below the limit of detection - for up to one year after the cessation of ART. These findings set the stage for future work to develop polyICLC-mediated augmentation of mucosally applied therapeutic vaccine approaches to help control HIV.

## Supporting Information

Figure S1 No correlation of T cell parameters with virus control (A) T cell subsets and activation markers (in the CD3+CD4+ and CD3+CD4− gates) were measured in PBMC, lymph nodes (LN), jejunum and ileum of all available animals at the time of necropsy (n = 6 in C274/AT-2 SIV group, n = 6 in polyICLC/AT-2 SIV group). Tregs were defined as CD25+FoxP3+; naïve T cells as CD95−; central memory T cells (TCM) as CD95+CD28+ and effector memory T cells (TEM) as CD95+CD28−. Average percentages for each group are shown (±SEM). (B) PD-1 surface expression (CD4+PD1+ and CD4−PD1+) was measured in PBMC and LN of all animals at the end of the study, by flow cytometry. Average percentages of PD-1 expressing cells per vaccination group are shown (±SEM).
(1.45 MB TIF)

Figure S2 Similar cytokine, chemokine and Treg marker expression in tissues, between groups. RNA was extracted from tissue cell pellets (10×106 cells/pellet). RT-PCR was performed for a range of cytokines and chemokines, as well as Treg markers. (A) Jejunum and LN cell RNA expression of cytokines and Treg markers. (B) Jejunum cell RNA expression of cytokines and chemokines. mRNA levels are shown in arbitrary units (AU) which were calculated by subtracting the threshold cycle (CT) number of the gene of interest from 100, then normalizing by dividing with (100-CT) of housekeeping gene β-actin, then multiplying by 100. Means (±SEM) of 6 C274/AT-2 SIV and 4 polyICLC/AT-2 SIV animals are shown for LN and 6 C274/AT-2 SIV and 3 polyICLC/AT-2 SIV animals for jejunum.
(2.46 MB TIF)
